# The Future of the Pig Industry After the Introduction of African Swine Fever into Asia

**DOI:** 10.1093/af/vfaa037

**Published:** 2020-10-30

**Authors:** Yonlayong Woonwong, Duy Do Tien, Roongroje Thanawongnuwech

**Affiliations:** 1 Department of Farm Resources and Production Medicine, Faculty of Veterinary Medicine, Kasetsart University, Kamphaeng Saen, Nakhon Pathom, Thailand; 2 Department of Infectious Diseases and Veterinary Public Health, Faculty of Animal Sciences and Veterinary Medicine, Nonglam University, HCMC, Vietnam; 3 Department of Pathology, Faculty of Veterinary Science, Chulalongkorn University, Pathum Wan, Bangkok, Thailand

**Keywords:** African swine fever, Asia, food security, pigs

**Implications**

- Rapid outbreaks and the spread of African swine fever across China and other countries have resulted in the huge loss of pig populations and sustainability of the global food supply chain.
- Because the most vulnerable and affected segment will be the small holders of the pig population, transformation of the pig industry to medium- and large-scale farms, together with better standardized production systems and biosecurity, will facilitate future survival of the industry and can contribute substantially to food security.
- During the pork shortage, a looming food crisis changed protein consumption behavior from meat to alternative protein sources.
- The ASF outbreaks in Asia might render the virus deleterious to the global pig industry associated with globalization. Travelers and international trading are the major carriers of a human-driven disease such as the ASF virus.

## Introduction

World demand for food is expected to increase due to population growth and change in consumption patterns. Available food supply at affordable prices is of importance for the sustainability of global food security. Agricultural sector plays a major role in food security by providing enough food for society. Agricultural productivity growth is necessary for both domestic use and exportation. However, the fluctuations of production volume in major exporting and importing countries can directly impact the world food balance. Normally, agricultural products in many regions are primarily for domestic consumption. However, there are a few countries that provide the large scale of the agricultural production to support exports.

Several factors affect the success of livestock production systems including sustainable resource management, evolving agricultural technology, financial management, and animal disease control (Thornton, 2010). Climate change and deforestation could impact the sources of water and food supply. Urbanization can also generate pressure not only on reduction in agricultural land but also the entire supply chains for agricultural production (FAO, 2011). Moreover, trading limitations and regulations may have a negative impact on farmers and the agricultural sector, contributing to the food insecurity (FAO, 2016).

Animal-based foods are considered complete and high-quality sources of major dietary proteins. As the demand of animal products increases, pork will continue as one of the main protein sources in Asia, and pig production will continue to be one of the major farm animals in the livestock subsector. Although production systems have been developed to improve production yields, animal disease outbreaks are still the greatest challenge on the pig production and pork protein security. In the last decade, major swine viral diseases such as porcine epidemic diarrhea and highly pathogenic porcine reproductive and respiratory syndrome have caused a direct impact on pig production leading to economic losses, especially in the low biosecurity farms (backyard to middle-scale farms) (Wang et al., 2016; Tian et al., 2007). It was not until August 2018 that African swine fever (**ASF**) has caused a major impact on pig production in China, and later in other neighboring countries, causing the worst economic crisis for the swine industry and the food security in the regions (Yun, 2020).

## The Pig Industry Before the ASF Outbreak

During the past three decades, pig production has changed rapidly from smallholders to an intensive industry in many developing countries. In the developed countries, large-scale production systems have achieved high levels of production performance and become the main type of pig farming systems. Therefore, only a few traditional forms of pig production survive in the developed countries. In developing countries, large- and medium-scale production systems have also been practiced. However, the largest group of pig population is still found in the traditional small-scale production systems or backyard farming (Huynh et al., 2007; Thanapongtharm et al., 2016). Type of farm scales in Thailand is classified as smallholders (<50 pigs) and large-scale farming systems. In 2018, Thailand’s Department of Livestock Development reported that the majority of swine household was smallholders (93.51%), while the 6.48% of large-scale farms were classified as small farms for 4.98% (50 to 500 pigs), medium farms for 1.37% (500 to5,000 pigs), and large farms for 0.13% (>5,000 pigs). The smallholders are classified into two types: backyards and commercial smallholders (Charoensook et al., 2013; Thanapongtharm et al., 2016). The backyards commonly have native pigs either for breeding or fattening, or both. Although the total swine population increased, the average number of the smallholders declined, whereas the number of pigs per household increased. According to Thailand’s Office of Agricultural Economics, modern and intensive pig production systems in Thailand have been growing at a rate of 8.90% per year during 2014 to 2018. It could be speculated that Thai swine production has moved to intensive production systems consisting of more than 75% of the pig population in the country since the smallholders could not compete with the industrialized farms in terms of the production costs and performances (Thanapongtharm et al., 2016).

The development of technology, knowledge, and innovation has resulted in opportunities to improve management, housing, breeding, and feeding methods. In addition, the performance parameters of sows and growing pigs have been available on a computerized recording system to maximize the potential of sow and herd productivity (Udomprasert et al., 1993). These factors have also been the driving forces of the production growth and export opportunities of pig industry in Asia, including Thailand. Over the past 30 yr, pigs weaned per sow per year have increased from 20 to 30 pigs in high-performance herds (Koketsu et al., 2017). The better performances could be due to the efficiency of the better genetic lines and management leading to increased herd productivity, including litter per sow per year, total born per litter, and born alive per litter (Koketsu et al., 2017).

In recent decades, massive growth in pig production in the Asia-Pacific region has been recognized, whereas pig numbers in the United States and European Union are increasing slowly or holding steady. Major pig production areas are in Asia (mainly in China possessing over 50% of the world pig population), the European Union, the United States, Brazil, and Russia (USDA, 2020). China has been known as the world’s largest pig production and pork consumption country. Therefore, Chinese pig production is for domestic consumption. In 2016, pork consumption in China was ~54.98 million tons, which required around 1.62 million tons of imported pork to supply the domestic demand (FAO, 2018). However, Rabobank estimated that pork imports in China increased to 2 million tons in the first half of 2019 because the Chinese pork production had dropped up to 55% from the ASF outbreak. Moreover, ASF outbreaks cause serious consequences for other high pork consumption countries in Asia, including Hong Kong (SAR-PRC), Japan, Republic of China (Taiwan), Malaysia, and Republic of Korea, which require massive pork imports to supply their domestic consumption.

## Spread of ASF in China and Its Impact

**Figure 1.**
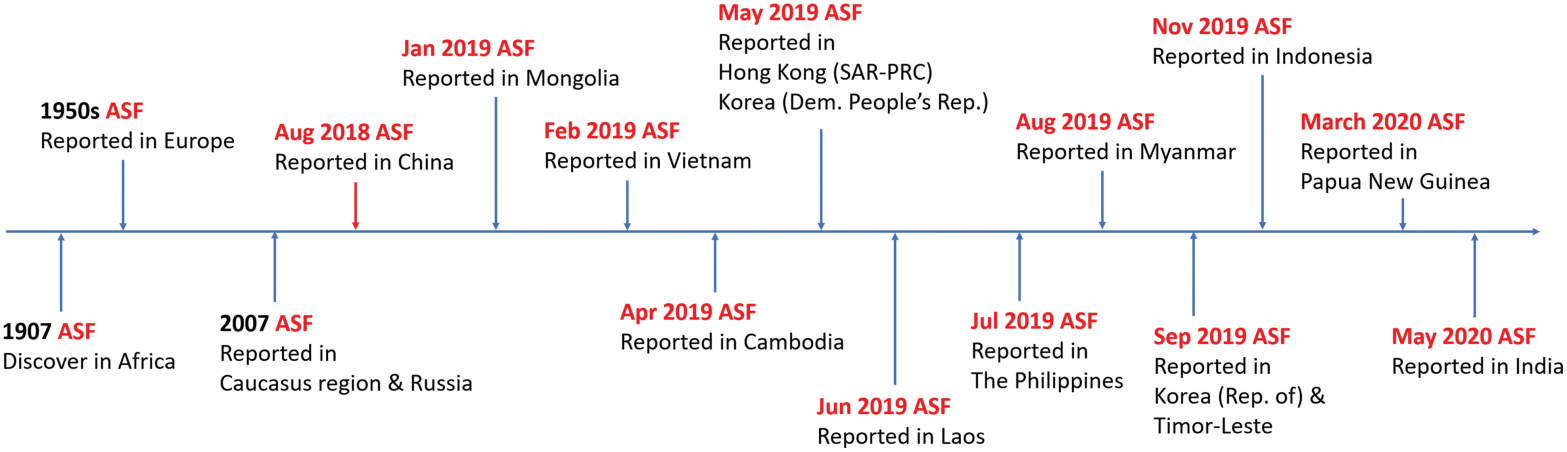
ASF outbreaks in Asia-Pacific region after the first outbreak in China.

ASF is one of the economically important viral diseases in the swine industry. Originating in Africa, ASF did spread across Europe through Georgia in 2007 (Sánchez-Cordón et al., 2018). The disease has gradually spread to other countries in the Caucasian region, and the Russian Federation, Eastern and Central Europe, and more recently Western Europe (Belgium). Since China confirmed the first case of ASF, the virus has already spread throughout the country within a few months and beyond its borders, unpredictably, across Asia. The disease continuously affects many pig production countries in Asia (Yun, 2020; Figure 1).

Starting from 2018 to early 2020, the outbreak of ASF has been reported in 21 countries in different regions around the world. In the Asia-Pacific region, the ASF outbreak has been found in at least 14 countries including Mongolia, Vietnam, Cambodia, Hong Kong (SAR-PRC), Korea (Dem. People’s Rep.), Laos, the Philippines, Myanmar, Republic of Korea, Timor-Leste, Indonesia, Papua New Guinea, and India (OIE, 2020).

**Figure 2.**
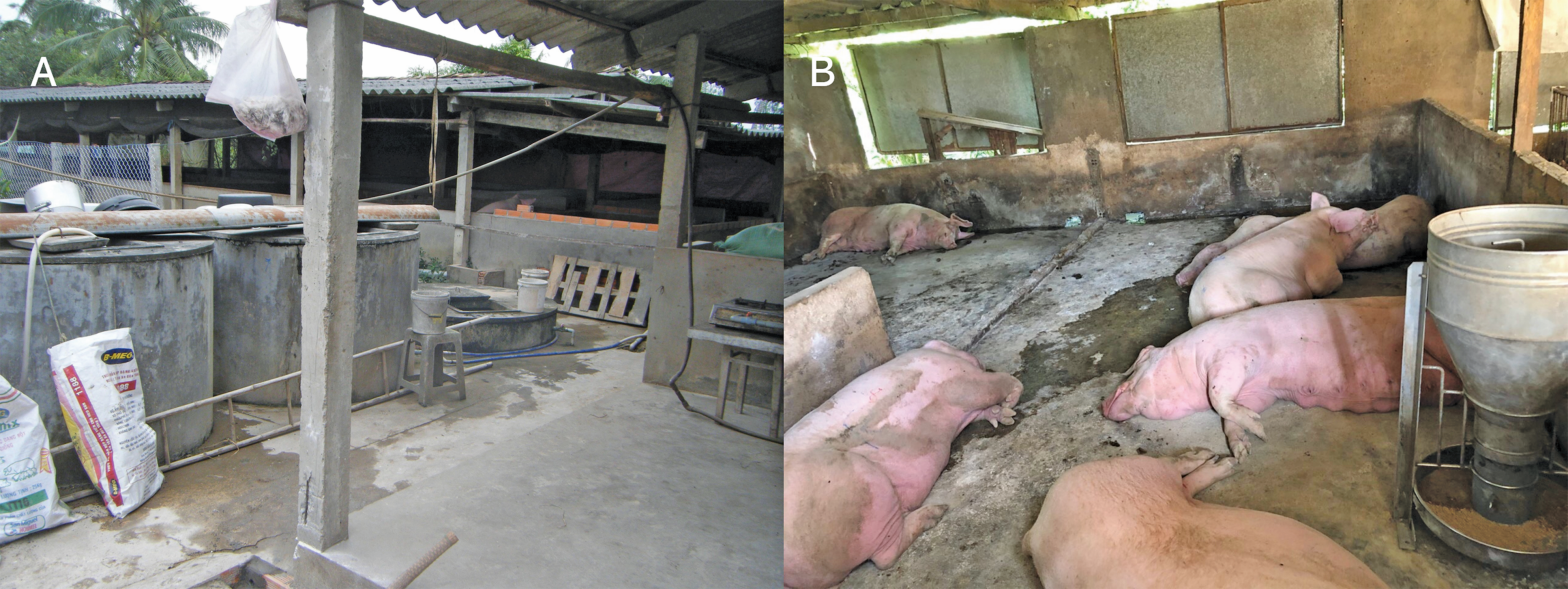
ASF outbreak in a typical small holder in Asia.

The Chinese government reported the outbreak of ASF in August 2018. Since the first ASF outbreak, more than 1 million pigs have been culled across China due to disease outbreaks in an attempt to stop ASF from spreading (Yun, 2020). Nevertheless, data showed that the outbreak resulted in a higher number of culled animals affecting directly to the small holders to be out of the pig business. Pig production loss was not only in the infected farms but also loss of producers whose farms were within the affected zones. Rabobank estimated that China’s pork production decreased by 25% in 2019 and by another 10% to 15% in 2020. A final prediction of as much as 700 million pigs lost would account for half the world’s pigs before the ASF outbreak. Furthermore, almost 70% of all outbreaks were found in the small herds having <50 animals (Figure 2). This is due to less awareness of the small holders regarding the implementation of proper biosecurity. Interestingly, ASF did spread across the whole country of China within 3 mo. Relevant factors in the spreading of ASF throughout the country included (1) the lack of live pig movement control, (2) insufficient capacity for rapid ASF detection, (3) inadequate animal quarantine, and (4) enforcement of transport bans (Wang et al., 2018). During the ASF outbreak, limited spaces for disposal of infected pigs and carcasses forced farmers in many areas to fail in the management of infected carcasses appropriately, resulting in the disposal of dead animals on roads, in the rivers, or in the forest. Moreover, this situation led farmers to sell their pigs to the slaughterhouses for quick cash. Therefore, tons of infected pigs were sold to market causing contaminated pork products entering the supply chains and spreading across China and the neighboring countries (Schulz et al., 2019; Yun, 2020).

During the past 10 yr, the fluctuations of pork prices depended on market forces. However, the ASF outbreak in China had a dramatic change in the fluctuation of pork prices. China’s pork production was massively disrupted, resulting in the highest pork prices ever and impacting to the market prices of other protein sources such as chicken meat and aquaculture products. Due to the increasing demand for pork in China, the control of live pig and pig product movement between regions in China is very difficult. Pork prices in China surged 47% in August 2019 (Yun, 2020). In addition, Rabobank estimated that pork prices could go even higher (Rabobank, 2019). China has attempted to supply the local demand by releasing large volumes of pork from state cold stores, but this has still not been enough. Consequently, declined domestic stocks and massive pig production loss in China significantly have had a major impact on global food security.

## ASF Penetration into the Vietnamese Swine Herds and Its Impact

In February 2019, ASF outbreak was first confirmed in a northern province of Vietnam by Vietnam Department of Livestock Production, Ministry of Agriculture and Rural Development (MARD, 2019). The ASF virus isolated belonging to genotype 2 was officially reported shortly after, with 100% similarity to the Chinese strain (Le et al., 2019). The occurrence of this ASF outbreak, though it may have been predicted a risk transmission couples of months earlier while the Chinese pig herds had been undergoing the ASF epidemics. Since ASF is an emerging disease, then the strategies and implementation of disease prevention and control from several local regulatory authorities, and pig farms were probably subjective with not enough experience and were unable to control the spreading. Trading, human travel between countries, movement of animals, and animal products are frequent and quite complicated to prevent the risk of ASF transmission and control. However, surveillance to prevent the entry of this virus into the territory requires a more comprehensive policy, laboratory and even a rapid response procedures and human resources for abnormal outbreak identification, testing and rapid actions to localize and confine the outbreaks. Meanwhile, the possibilities of ASF spread are through many routes such as roads, waterways, railways, and airways with associated with human activities and trading. The remarked feature of ASF virus is the long-term persistence and viability of the virus in contaminated fomites, foods, and feeds originated from infected pigs, which is an extremely favorable basis as a human-driven disease (FAO, 2017; Sánchez-Cordón et al., 2018; Mazur-Panasiuk et al., 2019).

**Figure 3.**
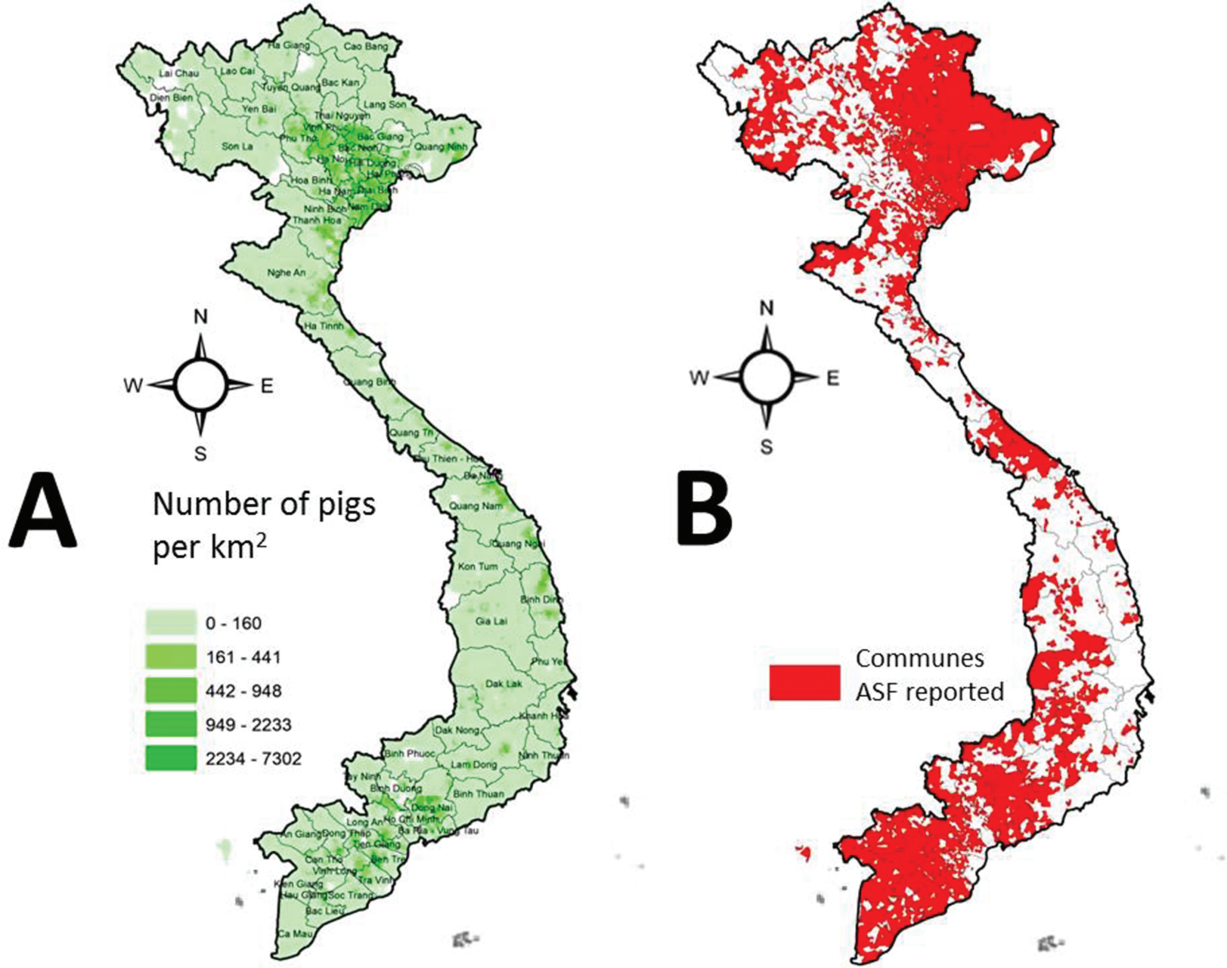
Estimated Vietnamese pig holding density (A) and ASF-affected communes in Vietnam (*Source*: Vietnam DAH, 2020).

Immediately after penetrating the Vietnamese pig herds, the spreading occurred quite rapidly. After a few months, the epidemic reached its peak, having spread to more than 8,200 communes in 63 provinces and cities within 9 mo (Figure 3). After 1 year, the loss officially reported due to the ASF epidemic was ~6 million head (accounting for 21.5% of the total herd), equivalent to the total pork weight loss of 342,091 tons (accounting for 9.0% of total pork production in the country). In Vietnam, pork production accounts for 71.5% of the livestock industry (in 2018) and is the main source of meat for local consumer diets. Therefore, the role of pork production is very important to the Vietnamese people and socio-economic activities associated with agriculture policies, food security, animal feed, veterinary, jobs, science and education, transportation, and other related activities. Comparing to the year before the ASF outbreak, the Vietnamese total pig herd and pork production volume decreased by 11.5% and 13.8%, respectively. The cumulative reduction in the total pig herd due to ASF immediately prompted the rapid growth of poultry production (16.5%), ruminant (over 5.0%), and other farm animals (over 3.0%) as well as rapidly increased pork imports in 2019 (63.0%).

Crisis shortages of pork were experienced when the ASF epidemic was at its peak. Directly infected pigs and pigs in the infected zones are culled to stop the spreading. The very low pork price differences in the epidemic zone compared with the higher pork prices in the disease-free areas are a great motivation for smuggling. Later, due to the shortage of pork throughout the country, price of pork were doubled or tripled. Many solutions have been applied such as restructuring the livestock sector, pork production model, centralized and controlled local supply chain and enhanced disease prevention by improving biosecurity at various levels to reduce ASF losses and reproduce pork supply capacity. Along with strengthening ASF control measures, ensuring food supply is promoted, such as shifting husbandry models and encouraging pig repopulation toward strict biosecurity, and at the same time increasing development of poultry flocks and ruminant herds. In the long-term solution, necessary conditions for re-stocking of pigs with high biosecurity poses a difficulty to the small to medium farms on finding the areas of disease free together with huge investment in qualified imported breeders and well-equipped farm facilities.

Repopulation is an early implementation strategy to prepare for the scenario of pork shortage. The success of restocking is questionable on proper disease control continuing to occur when latent circulation of the ASF virus still exists in the infected areas due to the virus persistence in the environment. Currently, ASF has become endemic in several pig production areas in Vietnam. Eliminating ASF virus from the pig herds or from the country in general is absolutely a big challenge and it may take decades. ASF prevalence and contamination of the virus in the environment could have threatened the repopulation of the small to medium farms and even the big industry farms with good biosecurity. In addition to ASF, the Vietnamese pig production has also been burdened with several major pathogens (Carrique-Mas and Bryant, 2013; Chu et al., 2019; Toan et al., 2019).

Practical researches are needed to clarify several major issues including suitable farming models in the ASF virus contaminated areas, proper procedures to test disease-free pigs for repopulation, risk analysis on the supply chain within and between countries, and inactivation procedures on contaminated water and feed. It should be noted that wild boars have not yet played a major role in the ASF transmission in Asian countries. However, it is worth finding if wild boars would be a part of the reinfection later in the infected countries. The establishment and implementation of technical training and education programs provided by FAO and local authorities to improve the stake holders’ knowledge and farming practice on ASF control are essential. Specialized technical teams should be established and deeply trained to be ready to guide, respond, and promptly handle disease consequences to ensure successful and sustainable restocking. Ultimately, research on vaccine and antiviral medicine development are more proactive strategy and expectations for the current disease control for the farmers in the infected countries.

## Key Factors of ASF Spreading in Vietnam

The spreading and persistence of ASF is often associated with traditional small-scale production systems, and usually the absence of disease prevention and control strategies (Li and Tian, 2018). The traditional farmers are the most challenging audience for disease control and prevention due to having lower levels of awareness, low biosecurity system, unavailability of funds, weak bargaining power, low compliance with regulation, and limited access to proper treatment. For these reasons, the small-scale production systems are at risk for ASF invasion into a country’s overall herd. Vietnam serves as an example of a country where spread through traditional small-scale production.

The Vietnamese ecosystem links people, animals, and the environment very closely, not only in terms of habitats but also in terms of health and emerging diseases. Vietnam is the world’s 15th most populous country. The density of people and terrestrial and underwater animals is high, particularly in the outskirt area of big cities. Vietnam is experiencing rapid growth of livestock production. In 2018, the total pig herd was officially estimated at 28 million, the 6th largest producer of pork in the world. The pig production increased in not only in large-scale farms but also in diverse farming models varying from household, backyard farm, concentrated farms (medium and large), and cooperatives. However, the actual estimated number of pigs in the whole country had actually eclipsed over 40 million in 2016 due to the rapid increase of small farms which were not yet fully determined. The small and scattered pig production still accounts for a high proportion, with a total of 2.9 million pigs estimated over 50% of pigs nationwide. This scenario creates a diverse and complex ecosystem for animals, people, and pathogens.

The spread trajectory of ASF in Vietnam is noted to be somewhat complicated. Earlier, the disease occurred in backyard and/or small farms because pigs are often fed or contact with salvaged food and leftovers from households and restaurants. If those pigs were not handled strictly and quickly diagnosed for quarantine and stamping out, the sick pigs were then sold to the local slaughterhouses and later the virus would widely spread into the environment and supply chain. Then, the local outbreaks began to spread through small farms, particularly, the opened-door farms having poor biosecurity and farming practices from villages to villages. Later, widespread contamination including the presence of ASF virus in the environment creating many possible vectors would eventually gain its presence into the larger farms. In addition, the long-standing tradition of purchasing fresh meat from Vietnamese wet markets or directly from the local slaughterhouses could facilitate the spreading of the virus locally. Additionally, there are huge numbers of pig farming combining with human houses to reuse the leftovers and food waste for feeding pigs in order to save cost of pig feed, leading to the higher risk of getting infected (Toan et al., 2019). Thus, the major reasons why ASF spreads rapidly and widely in the Vietnamese pig herds include inadequate surveillance and monitoring, proper protocol when found the outbreaks, farmer knowledge and cooperation with the authorities, limitation on ASF prevention works, knowledge and understanding on applying the biosecurity to backyard farms as well as living habits and activities of people together with the complexity of food supply chains.

ASF virus is characterized by slow and not high spread in nature when the infected pigs are strictly monitored, isolated, and fully quarantined (Guinat et al., 2014; Chenais et al., 2019), but the complexity leads to widespread and rapid transmission of virus in the role of humans known as “a human-driven disease” (Dixon et al., 2020), especially for highly complex pork food chains in the domestic pig sector in Asia. Resistance and long-term survival are of important factors in supporting virus transmission via the human vector and other fomites (FAO, 2017; Sánchez-Cordón et al., 2018; Petrini et al., 2019).

## ASF Contingency and Preparedness Plan in Thailand

A high risk of ASF introduction into Thailand is considered since the country is surrounded by the ASF-infected neighboring countries. Based on the risk assessment of ASF introduction into Thailand without emergency response plans, it could cause severe economic losses at least 125 billion baht (USD 4.045 billion) (Sontiphun, 2019). It should be concerned that the limited availability for restoring pigs and recovering occupations of the farmers might lead to food insecurity in the future if having ASF in the country.

**Figure 4.**
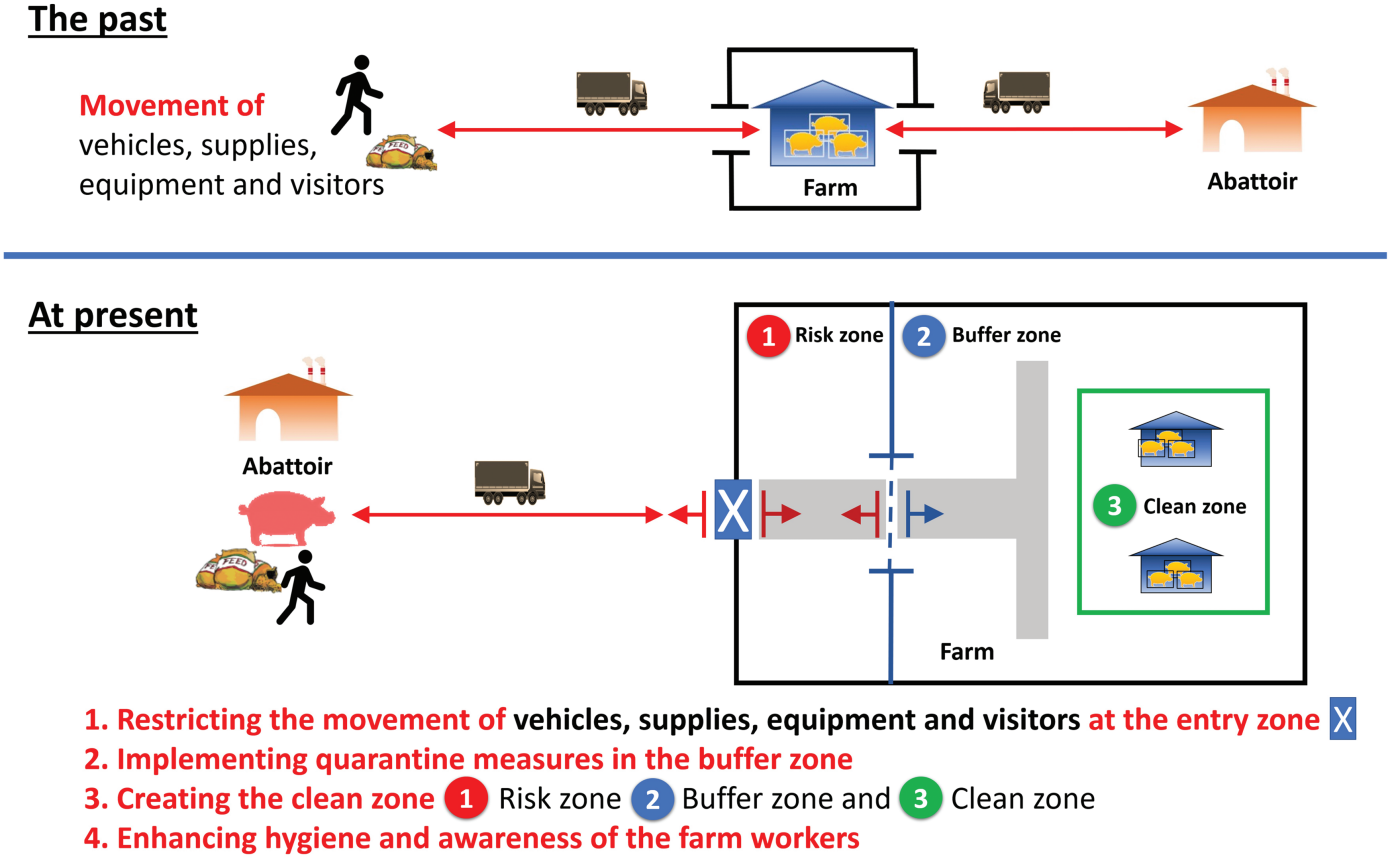
New normal practicing biosecurity when having ASF in the areas: (1) restricting the entry zone, (2) implementing quarantine measures in the buffer zone, (3) creating the clean zone, and (4) enhancing hygiene and awareness of the farm workers.

The Thai government approved 150 million baht (USD 4.7 billion) on the preparation for emergencies on national level. Accordingly, the cooperation between the Livestock Department with relevant agencies, swine farmers, and the private sectors has been involved for ASF contingency plans. This plan is comprised of three phases: pre-outbreak, outbreak, and postoutbreak and spending mainly for the risk factor control associated with the ASF introduction including (1) illegal movement of pigs and pork products along the border areas; (2) tourists and visitors from the ASF affected countries; and (3) vehicles, tools, equipment, pigs, food, and feed from the ASF risk areas. The development of disease diagnosis, establishment of laboratory networks, and increased public awareness is included. In addition, on-farm biosecurity standard has greatly been improved (Figure 4) by increasing investment in quarantine measures, workflow management, capacity building, employee hygiene, and disinfectant training. However, backyard farms might become the key risk in ASF outbreak. Thus, enhancing knowledge and awareness of local farmers particularly at the border areas are important for disease control in general.

## The Future of Pig Industry After the Introduction of ASF into Asia

**Figure 5.**
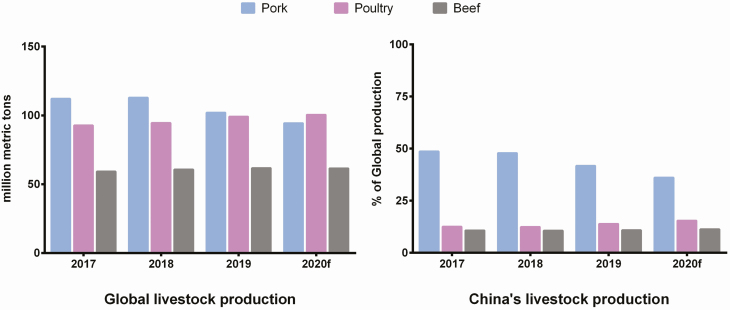
Global and China livestock production (carcass weight equivalent) (*Source*: USDA, 2020)

After ASF outbreak, the global food security will be challenged by a growing demand for agricultural products and the efficacy of productivity varied by regions. This current shortage in pork availability has impacted to the pork prices and changed in meat consumption behavior to other alternative protein sources. Rabobank showed that the global animal protein production was slow in 2019. It might be because of a dramatic decline in Chinese pork production while the other meat production was rising (Figure 5). European Union has become the top continents for exported pork products from 3.05 million tons in 2018 to 3.9 million tons in 2019 after ASF outbreak in Asia (USDA, 2020). The United States, Canada, and Brazil are the major pork exporters. Having the highest number of pigs in the country, China is still known as a net importer of pork. It is not only for pork. Beef are also imported mainly from South America, while poultry imports are also increased from Brazil and the European Union.

Furthermore, the impact of rising feed ingredient prices on costs of livestock production would impact livestock producers because 60% to 70% of pig production cost are feed cost. Therefore, the cost has a dramatic effect in small-scale farms comparing to the large-scale farms since the ability to organize the feed supply operations serving stores and the opportunity cost of labor and medicine do favor the big operation. Many traditional small scale and backyard farming would gradually disappear.

However, the ASF crisis would often lead to the potential investors to be major players in the future pig industry. ASF free herds or countries would gain greater benefit for exportation of pork. Therefore, the transformation of the pig industry from backyard and small scale to medium- and large-scale farms together with better standardized production systems and biosecurity would survive in the future.

At present, the pig production trend has changed to the medium- to large-scale production systems in many countries. However, the stakeholder network society of the pig industry in the region is still unable to control the pig movements within or between countries due to high demand in restocking and trading. The highly interconnectivity of worldwide pork production and globalization due to heavy trafficking of people could increase the risk of foreign pathogens introduction into other countries (Jurado et al., 2019; Kim et al., 2019). During the coronavirus infection disease 2019 (**COVID-19**) situation (Contini et al., 2020; Singhal, 2020), limitation of travelling within and among countries has had a positive effect on the control of ASF during the lockdown period. However, after all countries resume international travel post COVID-19 pandemic situation, ASF control strategies should not be neglected.

## Conclusions

Rapid outbreaks and the spread of ASF across China and other countries, resulting in the huge loss of pig populations and reflecting sustainability in the food supply chain. ASF outbreak has significant impact on the global protein supply, with concerning the balance of pork production and consumption. The soaring pork prices due to the shortage of pork have been increased in Asia. A looming food crisis has changed meat consumption behavior to other alternative protein sources. In any scenario, the most affected and most vulnerable segments of the pig population will be the small holders. Transformation of the pig industry to medium- and large-scale farms together with better standardized production systems and biosecurity would survive in the future. Additionally, improving productivity can also contribute substantially to food security. Therefore, the disease control strategies of various pig production systems in Asia are challenging. Since ASF has been known as a human-driven disease, the development of disease control measures and the collaboration with the relevant agencies and farmers could still be of importance in the ASF control strategies.

*Conflict of interest statement*. Authors have no conflicts of interest.

## About the Authors

Yonlayong Woonwong, a lecturer and researcher at the Faculty of Veterinary Medicine, Kasetsart University, Thailand. She received her DVM at Kasetsart University in 2009. She completed her Ph.D. in Veterinary Pathobiology at the Faculty of Veterinary Science, Chulalongkorn University, Thailand, in 2017. She had joined the Swine Production Medicine Clinical Rotation (SMEC 480) training at Iowa State University, in 2015. Her professional experience includes swine diagnosis, farm visits, and slaughter checks. Her research areas are focusing on swine disease control to improve the health and productivity.

Do Tien Duy (Assoc. Prof), a lecturer and researcher at the Faculty of Animal Sciences and Veterinary Medicine, Nonglam University, Vietnam. He received his DVM in 2004, his MSc of Veterinary Pathobiology at the Faculty of Veterinary Science, Chulalongkorn University, Thailand and his Ph.D. of Veterinary Medicine in SNU, Korea. He participated in many scientific researches in the field of virology and pathobiology of veterinary infectious diseases and in publishing books on pig diseases. He received several funded projects on veterinary/pig diseases and currently on ASF project funded by International Research Cooperation, Korean Government.

Thanawongnuwech (Prof.) is a native of Rayong, Thailand. He received his DVM in 1989 from Chulalongkorn University, Bangkok, Thailand and his Ph.D. (Vet Pathology) from Iowa State University (ISU) in 1998. He is currently working as a veterinary pathologist at Chulalongkorn University, focusing on emerging diseases in animals and concurrently, is an Affiliate Professor at the Department of Veterinary Diagnostic and Production Animal Medicine, College of Veterinary Medicine, ISU. He authors and coauthors over 100 scientific papers. Pathogenesis of swine viral pneumonia and other emerging diseases and development of diagnostic assays are of his interest.
